# Experimental Diabetic Retinopathy in Wistar Rats Induced by Streptozotocin: A Window into Retinal Disease Progression

**DOI:** 10.3390/ijms27083427

**Published:** 2026-04-11

**Authors:** Luis Fernando Barba-Gallardo, Manuel Enrique Ávila-Blanco, Javier Ventura-Juárez, Martín Humberto Muñoz-Ortega, Ruth Clarisa Murillo-Ruíz, Marcela Rivera-Delgadillo, José Luis Díaz-Rubio, Elizabeth Casillas-Casillas, Luis Héctor Salas-Hernández, Paloma Lucía Guerra-Ávila

**Affiliations:** 1 Departamento de Optometría, Centro de Ciencias de la Salud, Universidad Autónoma de Aguascalientes, Av. Universidad 940, Aguascalientes 20100, Mexico; fernando.barba@edu.uaa.mx (L.F.B.-G.); manuel.avila@edu.uaa.mx (M.E.Á.-B.); elizabeth.casillas@edu.uaa.mx (E.C.-C.);; 2 Departamento de Morfología, Centro de Ciencias Básicas, Universidad Autónoma de Aguascalientes, Av. Universidad 940, Aguascalientes 20100, Mexico; jventur@correo.uaa.mx; 3 Departamento de Química, Centro de Ciencias Básicas, Universidad Autónoma de Aguascalientes, Av. Universidad 940, Aguascalientes 20100, Mexico; humberto.munozo@edu.uaa.mx (M.H.M.-O.); clarisamurilloruiz@hotmail.com (R.C.M.-R.); mr9962703@gmail.com (M.R.-D.); 4 Patronato de Banco de Ojos y Tejidos de Aguascalientes, Av. Lic. Adolfo López M. Ote 1808, La Hacienda, Aguascalientes 20190, Mexico; joseluisdiazrubio@yahoo.com.mx

**Keywords:** murine model, diabetes *mellitus*, retinal disorder, retinal inflammation, gene expression, precision ophthalmology

## Abstract

Diabetic retinopathy (DR), recognized as a progressive neurovascular and microvascular complication of diabetes, remains one of the leading causes of visual disability worldwide, within the context of a sustained increase in ophthalmic diseases and retinal vascular disorders that compromise vision. This study aimed to characterize the progression of diabetic retinopathy in a streptozotocin (STZ)-induced Wistar rat model. A single dose of 65 mg/kg body weight was administered, with follow-up periods at 2, 4, 8, and 10 weeks, compared to healthy controls. STZ-induced rats exhibited reduced weight gain compared to the control group. They also showed markedly variable hyperglycemia, with glucose concentrations ranging from 250 to 530 mg/dL. Histological analysis of retinal tissue at week 4 revealed early signs of vascular compromise, including early indications of a microenvironment conducive to neovascularization and edema. By week 8, retinal damage had progressed to hemorrhage, persistent edema, and layer-specific vascular disruption. At week 10, intensified neovascularization and exacerbated edema indicated advanced microvascular deterioration. Immunofluorescence analysis demonstrated a temporal accumulation of CD8^+^ T cells in the retina, correlating with photoreceptor degeneration. The coordinated dynamics of CD4^+^ and CD8^+^ T cells suggested transient immune activation during STZ-induced retinal degeneration. Gene expression profiling revealed a proinflammatory and pro-oxidative retinal microenvironment, characterized by the overexpression of angiogenic pathways and proliferative signals. Simultaneously, the antioxidant response appeared partially impaired. Collectively, these findings provide mechanistic perspective on the multifactorial nature of diabetic retinopathy. Oxidative stress, inflammation, and angiogenesis converge to disrupt retinal homeostasis. This experimental model may serve as a reliable platform for future studies aimed at elucidating disease pathophysiology, identifying novel therapeutic targets, and evaluating emerging ophthalmic antidiabetic interventions.

## 1. Introduction

Diabetic retinopathy (DR) is a microvascular and neurodegenerative complication of diabetes *mellitus* (DM), affecting approximately 35% of people with diabetes worldwide, with significant variation across ethnic and geographic populations [1,2,3,4,5,6,7]. Within 25 years of diagnosis, over 95% of type 1 diabetic patients and around 60% of those with type 2 diabetes will develop some degree of DR [1,2,3,4,5,6,7,8,9,10,11]. DR remains the leading cause of preventable vision loss and blindness among adults aged 20 to 74, especially in middle- and high-income countries [1,2,3,4,5,6,7,9,10,11,12]. The onset and progression of DR are driven by chronic hyperglycemia and the accumulation of advanced glycation end products (AGEs), which disrupt normal metabolic pathways and trigger oxidative stress and early neurodegenerative changes in the retina [13,14,15,16]. In its initial stages, retinal swelling and the deposition of extracellular material occur, yet most patients remain asymptomatic. As the disease advances, damaged blood vessels leak fluid and blood into the retinal tissue, leading to significant visual impairment and, ultimately, blindness [7,10,11,12,13,14,15,16,17]. Common clinical manifestations include fluctuating vision, floaters and scotomas, corneal abnormalities and abrasions, diplopia, ocular pain, nystagmus, cataract formation, and distorted or blurred vision [3,7,17].

Global epidemiological data underscore the growing public health impact of DR. The Global Burden of Disease study identified DR as the fifth leading cause of both blindness and moderate-to-severe visual impairment in adults aged 50 and older [18,19]. Between 1990 and 2020, the age-standardized prevalence of blindness due to diabetic eye disease rose by 14.9% to 18.5%, and forecasts predict that nearly 191 million people worldwide will be living with DR by 2030 [20,21]. These trends highlight an urgent need for enhanced screening, early detection, and effective therapeutic strategies.

The pathogenesis of DR is multifactorial, involving molecular, biochemical, structural, and functional abnormalities that converge to damage both retinal neurons and vascular cells [22,23,24]. Central to this process is oxidative stress, a state in which reactive oxygen species (ROS) such as superoxide anion (O_2_^·−^), hydrogen peroxide (H_2_O_2_), peroxyl radicals (ROO·), and hydroxyl radicals (·OH) accumulate beyond the cell’s antioxidant capacity [25,26,27]. Hyperglycemia amplifies intracellular ROS production, and excessive ROS in turn injures the retinal microvasculature, leading to capillary basement membrane thickening through altered extracellular matrix turnover, breakdown of the blood retinal barrier (BRB), and formation of acellular, occluded capillaries, hallmarks of progressive DR [27,28,29].

DR is classified based on the presence or absence of neovascularization (NV). Pathological angiogenesis is the hallmark of proliferative diabetic retinopathy (PDR) [30], whereas its absence characterizes non-proliferative diabetic retinopathy (NPDR) [1,2,3,4,5,6,7,9,10,11,12,13,14,15,16,17,18,19,20,21,22,23,24,25,26,27,28,29,30,31,32,33,34,35,36,37,38,39,40].

Mild NPDR represents the earliest and most prevalent stage of DR. It is defined by the presence of microaneurysms (MAs), venous occlusions, intraretinal microvascular abnormalities, and subtle dilation of retinal vessels. These are early indicators of retinal damage [41,42]. NPDR encompasses a spectrum of changes triggered by chronic hyperglycemia. It begins with microangiopathy marked by pericyte loss and endothelial cell injury. These alterations lead to disrupted retinal blood flow, thickening of the retinal basement membrane (BM), direct macular damage (diabetic macular edema, DME), formation of acellular capillaries, MA development, venous beading, and dot-blot intraretinal hemorrhages. The release of vasoactive substances promotes NV [43,44,45,46]. Increased permeability of the BRB allows leakage of inflammatory cytokines and plasma proteins, resulting in hard exudates visible on fundus examination. As the disease progresses, vasoconstriction and capillary occlusion induce tortuous capillaries and retinal ischemia. This is often accompanied by cotton-wool spots [16,17,18,19,20,21,22,23,24,25,26,27,28,29,30,31,32,33,34,35,36,37,38,39,40,41,42,47].

PDR constitutes the most advanced stage of DR and is associated with severe vision loss. As DR progresses, new blood vessels begin to form on the retinal surface. These vessels are structurally unstable and prone to leakage of blood and extracellular fluids. In the final stages of PDR, profound hypoxia drives NV, vitreous hemorrhage, and retinal detachment [48,49]. Extensive ischemia, NV, and subsequent complications such as vitreous hemorrhage and tractional retinal detachment are characteristic of advanced PDR. These conditions often require surgical intervention [1,2,3,4,5,6,7,9,10,11,12,13,14,15,16,17,18,19,20,21,22,23,24,25,26,27,28,29,30,31,32,33,34,35,36,37,38,39,40,41,42,43,44,45,46,47]. Interestingly, PDR progression stabilizes in about 60% of patients, even among those with DM for over 50 years [1,2,3,4,5,6,7,8,9,10,11,12,13,14,15,16,17,18,19,20,21,22,23,24,25,26,27,28,29,30,31,32,33,34,35,36,37,38,39,40,41,42,43,44,45,46,47,48,49,50]. This suggests the existence of endogenous protective mechanisms that may delay or prevent transition to PDR [51].

Mitochondrial quality control in the diabetic retina is critically dependent on balanced mitophagy, which selectively removes depolarized or damaged mitochondria through the accumulation of PTEN-induced putative kinase protein 1 (PINK1) on the outer mitochondrial membrane and the recruitment of Parkin. Parkin-mediated ubiquitination of mitochondrial proteins enables recognition by autophagy receptors binding to light chain 3 (LC3) protein, thereby directing them toward lysosomal degradation [52]. Dysregulation of PINK1/Parkin-dependent pathways, as well as receptor-mediated mechanisms such as BNIP3/NIX and fun 14 domain-containing protein 1 (FUNDC1), under hyperglycemic conditions disrupts mitochondrial fusion-fission dynamics [53]. This imbalance leads to mitochondrial fragmentation, mtDNA damage, and excessive mtROS generation in retinal endothelial cells (REC), retinal pigment epithelium (RPE), Müller cells, and ganglion cells [52,53]. Alterations in the PINK1/Parkin pathway within retinal microvascular endothelial cells and RPE promote the accumulation of dysfunctional mitochondria, sustaining oxidative stress, activating the NLRP3 (NOD-, LRR-, and pyrin domain-containing protein 3) inflammasome [52], and driving the release of proinflammatory cytokines IL-β and IL-18 [53]. These mediators increase vascular permeability, compromise tight junction proteins, and impair BRB integrity, thereby contributing to diabetic neurovascular injury [52,53]. Conversely, excessive mitophagy has been documented in REC and Müller cells under hyperglycemic states. This process, characterized by dynamin-related protein 1 (Drp1) driven fragmentation and thioredoxin-interacting protein (TXNIP) activation, induces hyperactive Parkin-dependent mitophagy, leading to mitochondrial depletion, apoptosis, and gliosis, ultimately weakening the BRB structure [53]. Collectively, both insufficient and excessive mitophagy compromise mitochondrial quality control. In either scenario, damaged mitochondria persist or cellular bioenergetics collapse, resulting in the overproduction of ROS, NLRP3 activation, cytokine release, and the BRB breakdown [52,53].

Current therapeutic strategies aim to counteract vascular endothelial changes in DR, including excessive angiogenesis, increased permeability, retinal inflammation, and elevated intraocular pressure (IOP) [54]. Clinical success has increasingly focused on targeting the molecular drivers of microvascular abnormalities. A pivotal breakthrough was the identification of vascular endothelial growth factor (*VEGF*) as a key mediator in DR. Elevated *VEGF* levels promote aberrant NV by signaling retinal endothelial cells, altering vascular permeability, and driving angiogenesis. Moreover, *VEGF* serves as a biomarker of microangiopathy in PDR [55,56]. Multiple multicenter clinical trials have demonstrated that anti-vascular endothelial growth factor (anti-VEGF) antibodies effectively reduce DME, prevent further vision loss, and in some cases, improve visual acuity [56,57,58].

Additional therapeutic approaches include intravitreal corticosteroids, laser photocoagulation, which has long been an effective method for controlling proliferation and edema, and vitrectomy, tailored to disease stage and pathology [59]. The treatment of many DR complications continues to be grounded in the use of Anti-VEGF agents and corticosteroids. However, a significant proportion of patients (approximately 40%) exhibit DME that is resistant to monotherapy, and a smaller subset (approximately 8%) remains refractory to dual therapy, resulting in suboptimal visual outcomes [60].

Therefore, the objective of this study is to investigate the molecular mechanisms associated with tissue damage and protection in diabetic retinopathy using a murine model. The objective is to establish the foundation for future therapeutic studies that will enable patients to adopt the most effective treatments and enhance their quality of life.

## 2. Results

### 2.1. Biological Parameters

#### 2.1.1. Streptozotocin-Induced Attenuation of Weight Gain in Wistar Rats in Early-Onset and Temporally Stable

The impact of streptozotocin (STZ) administration on the body weight (BW) of Wistar rats was systematically evaluated. Body weight was recorded at baseline and at the conclusion of the experimental period, and weight gain was calculated as the difference between these two measurements. A one-way analysis of variance (ANOVA) was performed to compare weight gain across groups, using a significance *p* ≤ 0.05.

**Figure 1:**
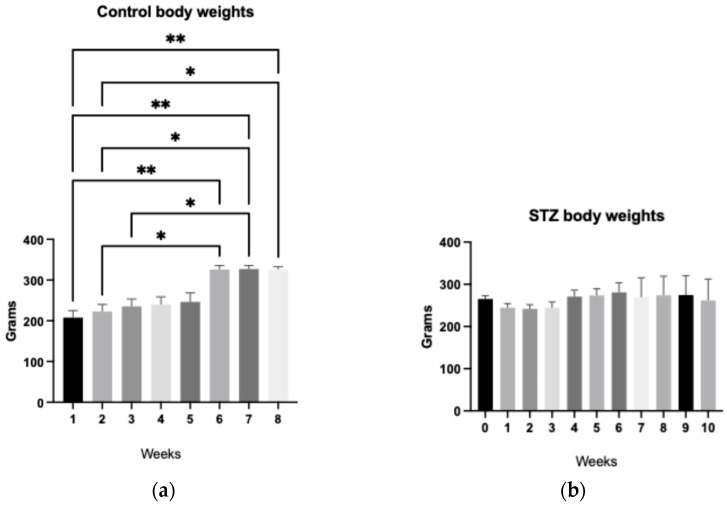
(**a**) Weekly body weight of control rats (citrate-buffered saline, *n* = 5), monitored up to 8 weeks. (**b**) Weekly body weights of streptozotocin-induced rats (STZ in citrate-buffered saline, *n* = 5), monitored up to 10 weeks. (ANOVA; * *p* ≤ 0.05, ** *p* ≤ 0.01).

Figure 1 illustrates the weekly progression of BW and cumulative weight gain for each group. Rats in the control group exhibited a significantly greater increase in BW compared to those in the STZ-treated groups. Among the experimental groups, no statistically significant differences in weight gain were observed across the various follow-up durations (2, 4, 8, and 10 weeks).

These findings indicate that STZ administration markedly attenuates weight gain in rats relative to the untreated control group, consistent with the metabolic disturbances typically associated with STZ-induced diabetes. The absence of temporal variation in weight gain among STZ-treated groups suggests that the effect of STZ on body mass stabilizes early in the disease course and remains relatively constant thereafter.

#### 2.1.2. Streptozotocin-Induced β-Cell Cytotoxicity Triggers Sustained Hyperglycemia in Wistar Rats

Blood glucose concentrations were measured in both control and STZ-induced rats to assess the metabolic impact of streptozotocin administration. Control animals maintained stable glycemic levels throughout the study, consistently below the basal threshold of 100 mg/dL, indicating normal glucose homeostasis.

**Figure 2:**
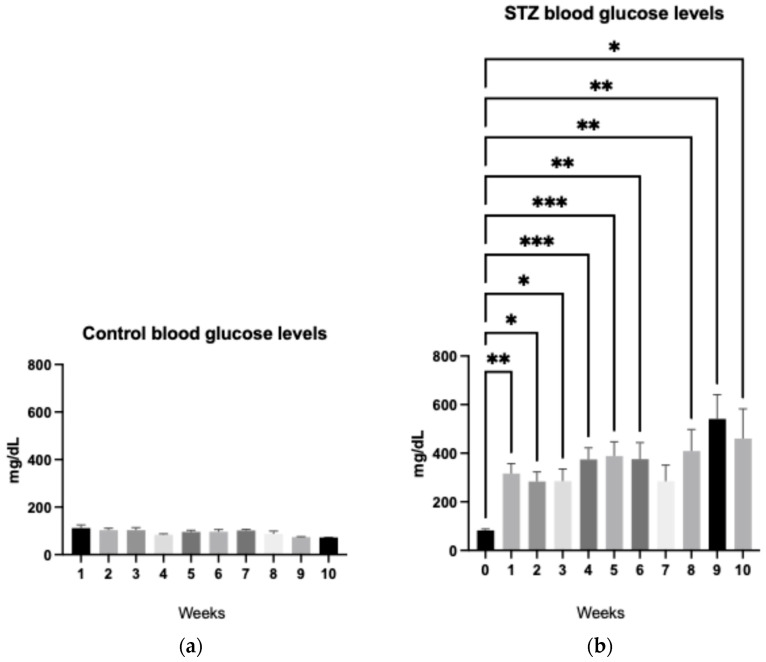
Blood glucose monitoring in experimental groups. (**a**) Weekly blood glucose in control rats given one dose of citrate buffer, tracked for 10 weeks. (**b**) Weekly blood glucose in streptozotocin-induced rats, monitoring over 10-week post injection (ANOVA; * *p* ≤ 0.05, ** *p* ≤ 0.01, *** *p* ≤ 0.001).

In contrast, rats injected with STZ exhibited a statistically significant elevation in blood glucose compared to their pre-treatment baseline values. Post-induction glycemia exceeded 250 mg/dL in all STZ-induced animals, with peak values reaching approximately 530 mg/dL, as illustrated in Figure 2.

These results confirm the successful induction of hyperglycemia and validate the STZ model for mimicking diabetic conditions. The marked increase in glucose levels reflects the cytotoxic effect of STZ on pancreatic β-cells, leading to impaired insulin production and sustained glycemic dysregulation.

### 2.2. Histology

#### 2.2.1. Preserved Retinal Architecture in Control Rats Confirms Baseline Histological Integrity

**Figure 3:**
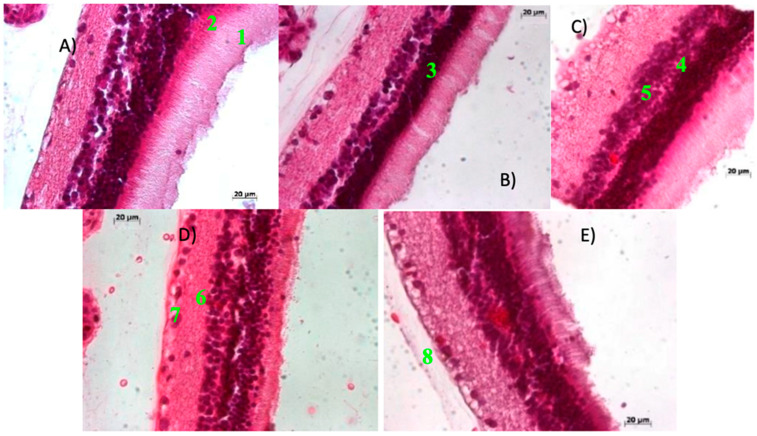
Histological evaluation of retinal tissue in control rats. (**A**) rat 1, (**B**) rat 2, (**C**) rat 3, (**D**) rat 4, and (**E**) rat 5. Representative photomicrographs of retinal sections stained with hematoxylin and eosin (H&E, 20× objective lens), obtained two weeks after administration of a single dose of citrate-buffered saline.

Figure 3 illustrates the retinal architecture of five control rats: (A) rat 1, (B) rat 2, (C) rat 3, (D) rat 4, and (E) rat 5. The following retinal layers were clearly identified in all specimens: (1) photoreceptor layer (rods and cones), (2) inner limiting membrane, (3) outer nuclear layer, (4) outer plexiform layer, (5) inner nuclear layer, (6) inner plexiform layer, (7) ganglion cell layer, and (8) optic nerve fiber layer.

No structural alteration or signs of damage were observed in any of the retinal layers across the control groups, indicating preserved histological integrity and normal retinal morphology under baseline conditions. These findings establish a reliable reference for comparative analysis with STZ-induced animals and support the validity of the control model.

#### 2.2.2. Week 4 Post-Streptozotocin: Retinal Early Indications of a Microenvironment Conducive to Neovascularization and Edema Reveal Early Vascular Disruption

**Figure 4:**
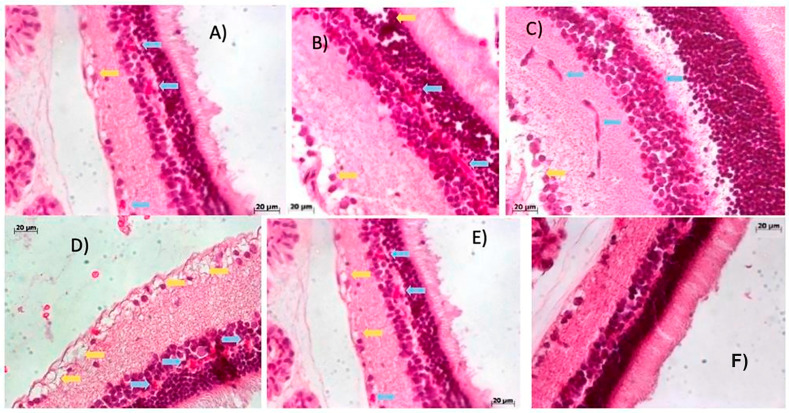
Histopathological alterations in retinal tissue from the streptozotocin-induced group. (**A**) rat 1, (**B**) rat 2, (**C**) rat 3, (**D**) rat 4, (**E**) rat 5 and (**F**) control group. Representative transverse sections of rat retina stained with hematoxylin and eosin (H&E, 20× objective lens), obtained four weeks after a single intraperitoneal dose of streptozotocin.

Figure 4 highlights pathological changes in retinal sections from STZ-induced rats at week 4. Blue arrows indicate early signs of a microenvironment conducive to neovascularization and the presence of erythrocytes. Yellow arrows mark regions of edema. These features appear in panels (A) through (E), corresponding to rats 1 through 5, respectively, alongside a control image for comparative reference.

A direct comparison between STZ-induced animals and the control group reveals distinct retinal damage induced by streptozotocin. Notably, panels (B) and (C) exhibit abnormal vascular proliferation: in panel (B), blood vessels are evident within the outer plexiform layer, whereas in panel (C), they appear in the inner plexiform layer, both with relatively preserved structural integrity.

Panel (D) demonstrates more pronounced pathological alterations, including marked edema in the ganglion cell layer and the presence of erythrocytes. These changes extend into adjacent layers, compromising their histological integrity. The widespread distribution of erythrocytes across multiple retinal layers, coupled with localized edema, underscores the severity of vascular disruption and tissue remodeling following STZ administration.

#### 2.2.3. Progressive Retinal Damage at Week 8: STZ-Induced Hemorrhage, Edema, and Layer-Specific Vascular Disruption

**Figure 5:**
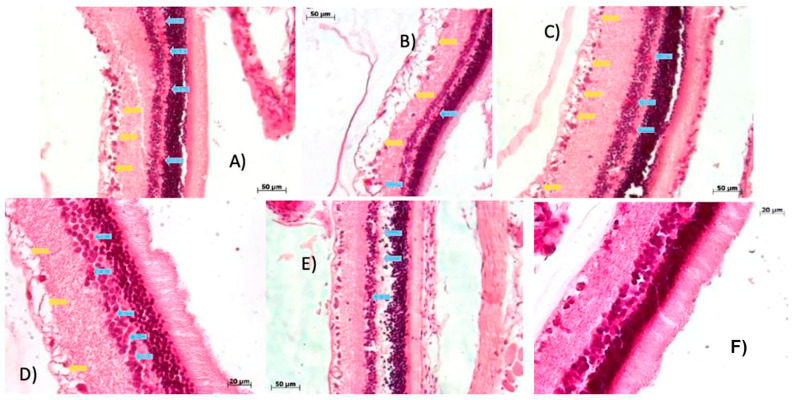
Histopathological changes in retinal tissue from the streptozotocin-induced rats. (**A**) rat 1, (**B**) rat 2, (**C**) rat 3, (**D**) rat 4, (**E**) rat 5 and (**F**) control group. Representative photomicrographs of transverse retinal sections stained with hematoxylin and eosin (H&E) 20× and 40× objective lenses, collected eight weeks after a single intraperitoneal administration of streptozotocin.

Figure 5 presents a comparative analysis of retinal alteration induced by STZ at 8 weeks post-injection, alongside a control image for reference. Blue arrows indicate regions of neovascularization and the presence of erythrocytes associated with hemorrhagic events, as observed in panels (A) through (E), corresponding to rats 1 through 5. Yellow arrows highlight areas of edema in panel (A), which were most prominent in panels (B), (C), and (D).

Panel (B) exhibited the highest concentration of erythrocytes within the ganglion cell layer, suggesting localized vascular disruption and hemorrhage. In contrast, panel (E) showed a greater accumulation of erythrocytes within the plexiform layer, although no edema was detected in that region.

Quantitative analysis revealed a 35% increase in edema within the ganglion cell layer and a 28% rise in erythrocyte presence across the nuclear and ganglion layers, relative to control values. These findings underscore the progressive nature of STZ-induced retinal damage, characterized by vascular leakage, tissue swelling, and compromised structural integrity, particularly in layers critical for visual signal transmission.

#### 2.2.4. Week 10 Post-Streptozotocin: Intensified Retinal Edema and Neovascularization Signal Advanced Microvascular Damage

**Figure 6:**
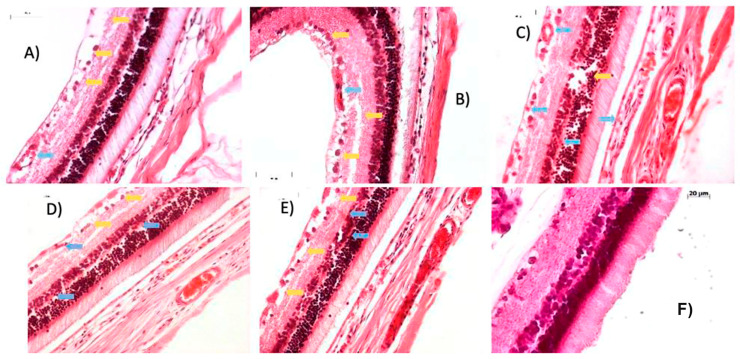
Histopathological assessment of retinal tissue in streptozotocin-induced rats. (**A**) rat 1, (**B**) rat 2, (**C**) rat 3, (**D**) rat 4, (**E**) rat 5 and (**F**) control group. Representative photomicrographs of transverse retinal sections stained with hematoxylin and eosin (H&E), captured at 20× objective lens, ten weeks after a single intraperitoneal dose of STZ.

Figure 6 illustrates the retinal effects of STZ at 10 weeks post-induction, compared to a control group. Blue arrows denote areas of neovascularization and erythrocyte accumulation due to hemorrhage, as observed in panels (A) through (E), corresponding to rats 1 through 5. These panels reveal a marked increase in erythrocyte infiltration and vascular proliferation within the inner retinal layers, particularly in the ganglion cell layer, where vessels appeared notably enlarged.

Yellow arrows indicate regions of edema, predominantly visible in panels (A), (B), (C), and (D). Panel (B) exhibited the most pronounced edema, characterized by significant separation within the ganglion cell layer that extended into the nuclear layers, a pattern also evident in panel (E), despite the absence of yellow arrow annotation.

Collectively, these findings confirm a substantial increase in edema affecting multiple retinal layers, accompanied by intensified erythrocyte presence and neovascularization within the ganglion cell layer. This progression underscores the severity of STZ-induced microvascular damage and its impact on retinal architecture, suggesting a sustained inflammatory and angiogenic response beyond the early stages of diabetic retinopathy.

### 2.3. Immunofluorescence

#### 2.3.1. Intrinsic M-Iodopsin Fluorescence and Hoechst Staining Confirm Retinal Integrity in Control Samples

**Figure 7:**
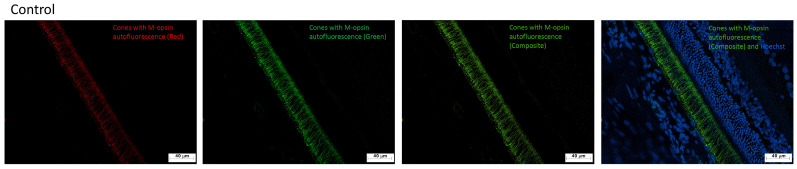
Immunofluorescence photomicrographs of retinal tissue from control rats. Cone photoreceptors exhibiting autofluorescence at 530 nm, corresponding to M-opsin (green), with an approximate emission peak at 554 nm.

Figure 7 displays the rod photoreceptors in retinal samples from control rats, which contain M-iodopsin, a naturally autofluorescent opsin. The intrinsic fluorescence of M-iodopsin enabled clear visualization of the rod outer segments without the need for additional labeling.

Furthermore, nuclear staining with Hoechst dye confirmed the structural integrity of the retinal layers. The distinct separation and organization of each layer were preserved, allowing for precise identification of nuclear positioning and interlaminar spacing. These findings validate the morphological preservation of the control retina and establish a baseline for comparison with STZ-induced animals’ samples.

#### 2.3.2. CD4^+^ T Cell Infiltration and Photoreceptor Degeneration Reveal Inflammatory Retinal Remodeling Post-Streptozotocin

**Figure 8:**
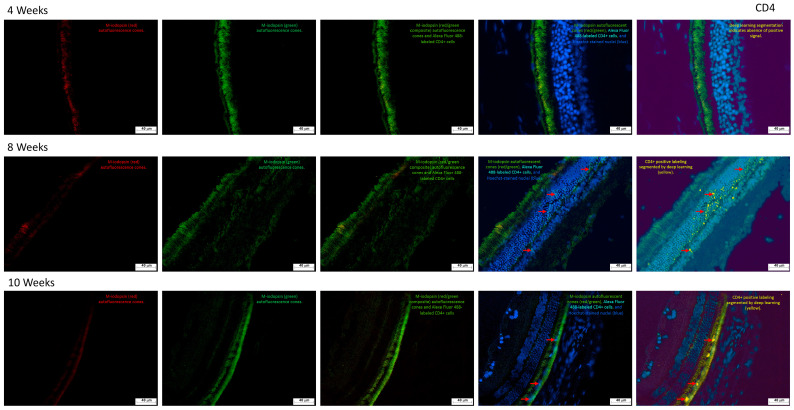
Immunofluorescence analysis of retinal tissue from streptozotocin-induced rats. Representative photomicrographs of transverse retinal sections collected at 4, 8, and 10 weeks following a single intraperitoneal dose of streptozotocin, visualized at 20× objective lens. A rabbit-derived anti-CD4 antibody was used as the primary marker to detect helper T lymphocytes, followed by Alexa Fluor 488-conjugated secondary antibody for fluorescence detection.

As shown in Figure 8, CD4^+^ helper T cells were detected using a rabbit-derived anti-CD4 primary antibody, followed by Alexa Fluor^®^ 488 as the secondary marker. CD4^+^ cells are indicated by red arrows in the images.

The intrinsic autofluorescence of M-iodopsin enabled visualization of the photoreceptor layer, allowing assessment of structural damage over time. Progressive deterioration of the rods and cones was evident across experimental weeks, highlighting the cumulative impact of STZ-induced retinal stress.

CD4^+^ cells were predominantly localized within the nuclear and plexiform layers, suggesting targeted immune infiltration in regions critical for synaptic transmission and cellular organization. These findings support the hypothesis of an inflammatory response contributing to retinal remodeling and functional impairment in diabetic conditions.

#### 2.3.3. Temporal Accumulation of CD8^+^ T Cells Correlates with Photoreceptor Degeneration in Streptozotocin-Induced Retinopathy

**Figure 9:**
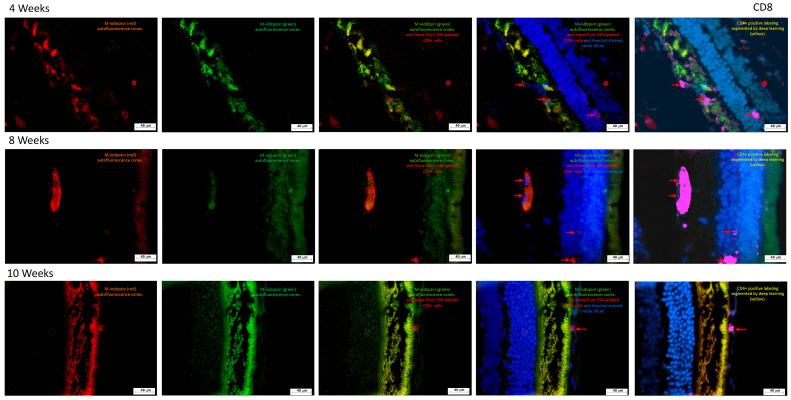
Immunofluorescence analysis of retinal tissue from streptozotocin-induced rats. Representative photomicrographs of transverse retinal sections collected at 4, 8, and 10 weeks after a single intraperitoneal dose of streptozotocin, visualized at 20× objective lens. A rabbit anti-CD8 antibody was used as the primary marker to detect cytotoxic T lymphocytes, followed by Alexa Fluor 594-conjugated anti-rabbit secondary antibody. CD8^+^ cells are indicated by red arrows in the merged image.

As illustrated in Figure 9, cytotoxic CD8^+^ T cells were identified using a rabbit-derived anti-CD8 primary antibody, followed by Alexa Fluor^®^ 594-conjugated anti-rabbit secondary antibody. In the merged image, CD8^+^ cells appear in red.

The autofluorescence of M-iodopsin enabled assessment of the structural integrity of the photoreceptor layer, revealing progressive degeneration of rods and cones throughout the experimental timeline. Notably, CD8^+^ T lymphocytes were first detected at week 4, with a marked increase in signal intensity by week 8.

These findings suggest a time-dependent infiltration of cytotoxic T cells into the retinal tissue, potentially contributing to the exacerbation of neuroinflammatory damage and photoreceptor loss. The spatial and temporal dynamics of CD8^+^ cell accumulation underscore their possible role in the pathophysiology of STZ-induced retinal degeneration.

#### 2.3.4. Coordinated CD4^+^ and CD8^+^ T Cells Dynamics Reveal Transient Immune Activation During Streptozotocin-Induced Retinal Degeneration

**Figure 10:**
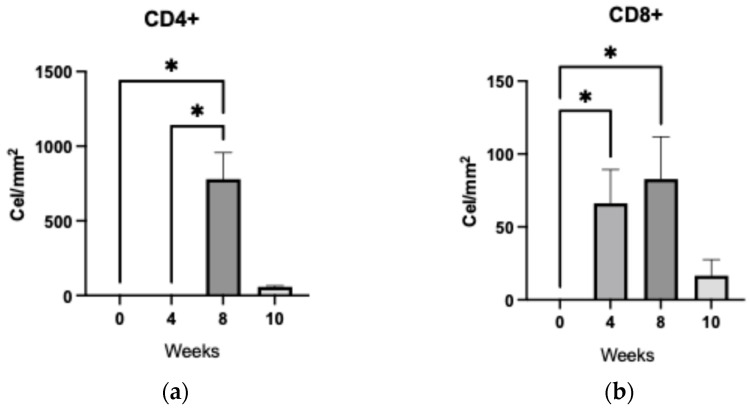
Quantification of T lymphocytes in rat retina. Graph (**a**) shows CD4^+^ cell counts and graph (**b**) shows CD8^+^ cell counts in retinal tissue from control rats at 10 weeks following a single dose of citrate buffer, and from streptozotocin-induced rats at 4-, 8-, and 10-weeks post-injection. Statistical analysis was performed using one-way ANOVA (* *p* ≤ 0.05).

Figure 10 illustrates the temporal dynamics of CD4^+^ and CD8^+^ T lymphocyte counts over a 10-week period. As shown in Graph 10a, CD4^+^ T cells exhibited a pronounced peak at week 8, reaching approximately 750 cells/mm^2^, followed by a sharp decline to 100 cells/mm^2^ by week 10. In contrast, CD8^+^ T cells (graph 10b) displayed a more variable pattern, with an initial count of 60 cells/mm^2^ at week 4, a modest rise to 80 cells/mm^2^ at week 8, and a subsequent decrease to 30 cells/mm^2^ at week 10.

A concurrent increase in both T cell subsets was observed at week 8, with the surge being markedly higher in the CD4^+^ population. This synchronized elevation suggests a coordinated immune activation phase, potentially reflecting a peak in retinal inflammatory response. The subsequent decline in both populations may indicate resolution or exhaustion of the local immune activity, aligning with the progression of retinal degeneration.

#### 2.3.5. Gene Expression Profile in Retinal Tissue of Streptozotocin-Induced Animals

The gene expression profile indicates a proinflammatory and pro-oxidative retinal microenvironment in STZ-induced rats, characterized by the activation of angiogenic pathways (*Vegfa*) and proliferative signals (*Egf*). Concurrently, the antioxidant response appears to be partially compromised. The upregulation of *Sod2* and *Cat* suggests compensatory activation of endogenous antioxidant defenses in response to excessive production of reactive oxygen species (ROS). However, the downregulation of *Nfe2l2* may imply that *Sod2* and *Cat* are being upregulated through alternative mechanisms such as cytokine-mediated signaling or mitochondrial feedback rather than through coordinated transcriptional control by *Nfe2l2*.

**Figure 11:**
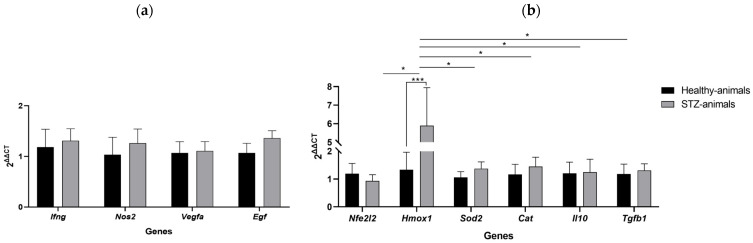
Analysis of oxidative stress and antioxidant gene expression in rat retina following streptozotocin administration. The relative expression of genes associated with oxidative stress (*Ifng*, *Nos2*, *Vegfa*, *Egf*) and antioxidant defense (*Nfe2l2*, *Hmox1*, *Sod2*, *Cat*, *Il10*, and *Tgfb1*) was evaluated in retinal tissue ten weeks after a single intraperitoneal dose of streptozotocin. Gene expression was quantified by real-time PCR using β-actin mRNA as the internal reference (2^ΔCT^ method). Relative expression levels were calculated using the 2^ΔΔCT^ method, comparing STZ-induced rats to healthy controls. (**a**) Relative expression of oxidative stress-related genes. Significant differences were observed between the control and STZ groups. (**b**) Relative expression of antioxidant-related genes. Mean expression levels of each gene were significantly altered in STZ-induced rats compared to healthy controls. Data are presented as mean ± SD. Statistical analysis was performed using one way ANOVA followed by Tukey’s multiple comparison test (* *p* ≤ 0.05, *** *p* ≤ 0.001).

The overexpression of *Nos2* and *Hmox1* reflects a cellular response to nitrosative and oxidative stress, while the reduced expression of *Il10* may indicate suppression of anti-inflammatory signaling. Finally, the increased expression of *Tgfb1* could suggest activation of fibrogenic or immunomodulatory pathways, Figure 11. Collectively, these findings are consistent with the early pathophysiological features of diabetic retinopathy.

## 3. Discussion

### 3.1. Pharmacological Induction of Diabetes Mellitus

The pharmacological induction of pancreatic β-cell destruction remains the most widely employed method for modeling type 1-like diabetes *mellitus* in rodents. Streptozotocin (STZ), administered via a single intraperitoneal injection, reliably induces hyperglycemia at doses ranging from 150 to 200 mg/kg in mice and 30 to 80 mg/kg in rats [61]. In the present study, we administered STZ at a dose of 65 mg/kg body weight, a concentration well tolerated by Wistar rats and effective in inducing diabetes. The diabetic state was confirmed by alterations in glycemic control, vascular integrity, and immunological parameters.

Previous studies using Sprague-Dawley rats reported significant weight loss (28 ± 20 g) within the first 72 h following a 65 mg/kg STZ dose, whereas doses of 40 or 50 mg/kg did not exhibit significant changes in body weight [62]. Consistent with these findings, our STZ-induced Wistar rats maintained stable body weight throughout the experimental period. In contrast, control animals subjected to the same hypercaloric diet exhibited a statistically significant increase in body weight. Notably, STZ-induced rats showed a marked reduction in adipose tissue, with minimal or absent brown fat deposits at euthanasia, while control rats retained visible abdominal brown adipose tissue.

### 3.2. Pathophysiological Effects of Streptozotocin-Induced Diabetes Mellitus

STZ-induced diabetes leads to persistent hyperglycemia, with blood glucose levels exceeding 200 mg/dL within the first week and surpassing 400 mg/dL thereafter findings consistent with previous reports [62]. In our study, some animals reached glycemic values above 600 mg/dL, exceeding the detection limit of the glucometer (Figure 2).

Beyond systemic metabolic disruption, STZ-induced diabetes caused progressive retinal damage, including hemorrhage, fibrovascular proliferation, and detachment of the photosensitive layer. These findings are consistent with those reported by Moldovan [63]. The alterations observed between weeks 2 and 10 post-induction represent hallmark features of proliferative diabetic retinopathy (Figure 3, Figure 4, Figure 5 and Figure 6) [61].

### 3.3. CD4^+^ and CD8^+^ T Lymphocytes

Immunofluorescence analysis revealed increased infiltration of CD4^+^ and CD8^+^ T lymphocytes in the retina (Figure 8 and Figure 9), particularly in the photoreceptor and nuclear layers. Although less evident than histological sections, the presence of retinal injury was discernible and consistent with diabetic retinopathy. Comparative studies have shown a predominance of CD4^+^ over CD8^+^ cells in DR models, a pattern replicated in our findings [64]. This adaptive immune-mediated inflammation was especially pronounced in the inner plexiform, nuclear, and photoreceptor layers.

By week 4, erythrocytes were detected in the inner plexiform layer (Figure 4), likely originating from newly formed blood vessels, accompanied by edema in the ganglion cell layer. These features intensified by week 8 (Figure 5), with detachment of the ganglion cell layer. At week 10 (Figure 6), edema extended into deeper retinal layers, and neovascular structures became more prominent. These cumulative changes validate the model as a reliable representation of diabetic glaucoma. Importantly, CD4^+^ and CD8^+^ T cells were elevated not only in the retina but also in peripheral blood and lymphoid tissues [65]. CD4^+^ T cells, crucial for immune homeostasis, are upregulated during diabetes progression [66], while CD8^+^ cells contribute to retinal neovascularization and vascular leakage, driving pathological angiogenic in DR [65].

### 3.4. Molecular and Oxidative Mechanisms

Oxidative stress plays a central role in DR pathogenesis, exacerbating hyperglycemia-induced metabolic disturbances and triggering molecular cascades that compromise retinal integrity [67]. Our PCR data revealed proinflammatory and pro-oxidative retinal microenvironment in STZ-induced rats, marked by upregulation of *Nos2*, a key indicator of nitrosative and oxidative stress. These findings align with reports linking *iNOS* expression to diabetes duration and early vascular [68,69].

The down regulation of *IL-10* suggests impaired anti-inflammatory signaling, potentially favoring activation of M1 macrophages, microglia, and B cells, as observed in DR patient samples [70,71,72,73]. This inflammatory shift may further amplify oxidative damage and vascular dysfunction.

Angiogenic and proliferative pathways were also activated, as evidenced by increased *Vegfa* and *Egf* expression. *Vegfa*, a member of the cystine knot superfamily, promotes angiogenesis and vascular permeability under hypoxic and oxidative conditions [74,75,76]. Its upregulation in serum and ocular fluids correlates with leukocyte adhesion, *ICAM-1* expression, and neovascularization in proliferative DR [70,77,78]. *Egf* similarly enhances retinal cell proliferation and neovascular formation [73,79].

These angiogenic signals may be potentiated by PKC pathway activation, which increases endothelial permeability and *Vegfa* expression [51,80]. Moreover, hyperglycemia, ROS, cytokines, PKC, and AGEs activate *NF-κB*, a transcription factor regulating over 500 genes, including *VEGF*, *IL-1*, *IL-6*, *TNF-α*, *MCP-1*, *VCAM-1*, and *PDGF* [81,82,83].

Interestingly, while *Sod2* and *Cat* were upregulated suggesting a compensatory antioxidant response, the downregulation of *Nfe2l2* implies alternative regulatory mechanisms. Although *Nfe2l2* nuclear levels increase in STZ-induced diabetic retinas, its binding to the antioxidant response element (ARE4) is impaired under hyperglycemia, reducing *Gclc* expression and GSH synthesis [84,85,86]. Histone methylation at the *Gclc*-ARE4 locus may further hinder *Nfe2l2* binding, and this repression persists even after glucose normalization.

*Hmox1*, a stress-inducible enzyme with antioxidant, anti-inflammatory, and anti-apoptotic properties, was also upregulated. Its induction modulates macrophage polarization toward the anti-inflammatory M2 phenotype, inhibits dendritic cell maturation, and promotes Treg responses, thereby mitigating oxidative stress and inflammation [87]. *Hmox1* also protects against pyroptosis and ferroptosis, emerging form of programmed cell death implicated in diabetic complications.

Finally, increased *Tgfb1* expression may reflect activation of fibrogenic and immunomodulatory pathways, contributing to endothelial proliferation and neovascularization [73,79]. Conversely, *Il10* downregulation may impair *Vegfa* suppression, fostering a proangiogenic environment [88,89].

## 4. Materials and Methods

### 4.1. Animals

A total of 30 male Wistar rats (6–8 weeks old; 150–220 g body weight) were obtained from the Bioterium of the Universidad Autónoma de Aguascalientes. Animals were housed under controlled environmental conditions (24 ± 2 °C; 55.5 ± 5% relative humidity) with a 12 h light/dark cycle and had *ad libitum* access to standard chow and water. Following a 7-day acclimatization period, the rats were randomly assigned into five experimental groups: a healthy control group (*n* = 5), which received a single intraperitoneal (i.p.) injection of citrate buffer (vehicle), and four streptozotocin (STZ)-induced diabetic groups (*n* = 5 per group), monitored at 2, 4, 8, and 10 weeks post-induction (total *n* = 20). To ensure the integrity of longitudinal study and account for potential attrition, we induced diabetes in five additional rats. This precaution allowed us to maintain a complete *n* = 5 per group throughout the 10- week follow-up, despite the loss of four animals’ post-induction.

All procedures were reviewed and approved by the Research, Bioethics, and Biosecurity Committees of the Universidad Autónoma de Aguascalientes (protocol INV 798/2021) and conducted in accordance with the Mexican Official Standard for the Care and Use of Laboratory Animals (NOM-062-ZOO-1999; revised 2001).

### 4.2. Induction of Type 1 Diabetes Mellitus (STZ Model)

Type 1 diabetes *mellitus* was induced following a 12-h fasting period via a single i.p. injection of streptozotocin (STZ; 65 mg/kg body weight; cat. S0130-1G, Sigma-Aldrich, St. Louis, MO, USA) freshly dissolved in 0.1 M citrate buffer (pH 4.5) [90]. Control animals received an equivalent volume (200 µL) of citrate buffer without STZ. Diabetes was confirmed by measuring the fasting glucose concentration (glucose ≥ 200 mg/dL) 1 week after STZ administration.

At the end of each experimental period, animals were euthanized by intraperitoneal administration of sodium pentobarbital (1.5 mL/100 g body weight), using a double dose relative to that required for sedation. Collected tissues were fixed in 4% paraformaldehyde and processed for histological analysis [91].

### 4.3. Analytical Procedures

#### 4.3.1. Blood Glucose Monitoring

Blood glucose levels were measured using an Accu-Check Performa^TM^ glucometer (Roche Diabetes Care, Mannheim, Germany). Samples were collected in the morning via tail-tip puncture.

#### 4.3.2. Body Weight

Rats were weighed weekly throughout the experimental period to monitor changes in body mass.

#### 4.3.3. Histological Analysis

Histological evaluations included hematoxylin and eosin staining, as well as indirect immunofluorescence. Primary antibodies were used at a concentration of 1:500, specifically CD4 Monoclonal (cat. 11-0041-82, Invitrogen, Waltham, MA, USA) and CD8 (cat. MCA1576GA, Bio-Rad, Hercules, CA, USA). Secondary antibodies were applied at a concentration of 1:1000, including Alexa Fluor^®^ 488 (cat. A11008, Invitrogen, Waltham, MA, USA) and 596 (cat. A11005, Invitrogen, Waltham, MA, USA). Nuclear staining was performed using Hoechst dye (cat. 33258, Thermo-Fisher, Waltham, MA, USA). Image acquisition and analysis were conducted with Image-pro Plus Software Version 5.4.0.19 (Media Cybernetics, Inc., Rockville, MD, USA), employing a Cool Snap-pro CF camera mounted on a Axioskop 40 microscope (Zeiss, Oberkochen, Germany). 

#### 4.3.4. RNA Extraction and Quantitative Real-Time Polymerase Chain Reaction (qRT-PCR)

Total RNA was isolated from the retinal tissue of healthy and STZ-induced rats using Trizol Reagent (cat. 15596026, Invitrogen, Waltham, MA, USA), strictly following the manufacturer’s protocol. Next, 5 µg of RNA from each sample were reverse transcribed using the High-Capacity cDNA Archive Kit (cat. 4322171, Applied Biosystems, Foster City, CA, USA). The resulting cDNA was amplified by quantitative real-time PCR on the SDS 7300 system (Applied Biosystems). *β-actin* served as the endogenous control due to its constitutive expression as a housekeeping gene. All reactions were performed in duplicate. Gene expression levels of *Ifng*, *Nos2*, *Vegfa*, *Egf*, *Nfe2l2*, *Hmox1*, *Sod2*, *Cat*, *Il10*, and *Tgfb1* were normalized to *β-actin*, and relative expression changes were calculated using the 2^−ΔΔCT^ method.

#### 4.3.5. Data Analysis

Image processing and quantification were conducted using Fiji software version 2.16.0/1.5p (Fiji Is Just ImageJ, GNU General Public License) [92], with subsequent deep learning-assisted classification performed via Ilastik. Positive cells were counted per field to estimate cell density (cells/mm^2^). Comparison of gene expression between experimental and control groups was conducted using Student’s *t*-test or one-way analysis of variance (ANOVA), as appropriate. A *p*-value < 0.05 was considered statistically significant. Graphs and statistical analyses were generated using GraphPad Prism 9 for macOS.

## 5. Conclusions

Our findings underscore the multifactorial nature of diabetic retinopathy, wherein oxidative stress, inflammation, and angiogenesis converge to destabilize retinal homeostasis. The STZ-induced model employed in this study faithfully recapitulates key molecular and histopathological hallmarks of human DR, thereby providing a robust and reproducible platform for mechanistic investigations and therapeutic development.

### Future Directions

Critically, future investigations in the fields of metabolic diseases and ophthalmology would be enriched by:

Early confirmation of neovascularization through histological analysis using Isolectin B4 staining.

Characterization of molecular factors preceding mitophagy deregulation, including the PINK1/Parkin signaling pathway, NLRP3 inflammasome activation, TXNIP involvement, Drp1, Mfn1/Mfn2, VDAC1, ZO-1, the LC3-II/LC3-I ratio, Sirt3, as well as BNIP3/Nix and FUNDC1 non-canonical pathways.

Exploration of calcium overload, which may induce mitochondrial permeability transition (mPT) via the mPTP pore, thereby facilitating the release of cytochrome c and other mitochondrial components.

The integration of multi-omics technologies will enable the identification of novel therapeutic targets with greater specificity and reduced adverse effects, supporting the development of more precise treatment for diabetic retinopathy. In parallel, the incorporation of Optical Coherence Tomography (OCT) for structural assessments and of Optical Coherence Tomography Angiography (OCT-A) for vascular function will provide an additional clinical dimension. These non-invasive imaging modalities, by complementing molecular and histological analyses, will allow longitudinal monitoring of retinal alterations, thereby strengthening translational relevance and broadening the scope of potential clinical applications.

## Data Availability

The original contributions presented in this study are included in the article. Further inquiries can be directed to the corresponding author.

## Abbreviations

The following abbreviations are used in this manuscript:

DR	Diabetic retinopathy
STZ	Streptozotocin
ROS	Reactive oxygen species
O_2_^⋅−^	Superoxide anion
H_2_O_2_	Hydrogen peroxide
ROO⋅	Peroxyl radicals
⋅OH	Hydroxyl radicals
NV	Neovascularization
PDR	Proliferative diabetic retinopathy
NPDR	Non-proliferative diabetic retinopathy
MA	Microaneurysms
BM	Basement membrane
DMD	Direct macular damage
DME	Diabetic macular edema
BRB	Blood-retinal barrier
DM	Diabetes *mellitus*
IOP	Intraocular pressure
H&E	Hematoxylin and eosin
RNA	Ribonucleic acid
cDNA	Complementary deoxyribonucleic acid
PCR	Polymerase chain reaction
ANOVA	Analysis of variance
CD4	Cluster of differentiation 4, often called helper T cells
CD8	Cluster of differentiation 8, often called cytotoxic T lymphocytes
*Vegfa*	Vascular endothelial growth factor
*Egf*	Epidermal growth factor
*Ifng*	Interferon gamma
*Nos2*	Inducible nitric oxide synthase
*Nfe2l2*	Nuclear factor erythroid 2-related factor 2
*Hmox1*	Heme oxygenase-1
*Sod2*	Mitochondrial superoxide dismutase 2
*Cat*	Catalase
*Il10*	Interleukin-10
*Tgfb1*	Transforming Growth Factor-beta
PKC	Protein kinase C
AGEs	Advanced Glycation End-products
NF-κB	Nuclear Factor-kappa B
IL-1	Interleukin-1
IL-6	Interleukin-6
TNF-α	Tumor Necrosis Factor-alpha
MCP-1	Monocyte chemoattractant protein-1
VCAM-1	Vascular cell adhesion molecule-1
PDGF	Platelet-derived growth factor
GCLC-ARE4	Glutamate-cysteine ligase catalytic subunit-antioxidant response elements 4
PINK1	PTEN-induced putative kinase protein 1
BNIP3	Bcl-2 and adenovirus E1B 19-kDa interacting protein 3
Nix	BNIP3-like protein (BNIP3L, also known as Nix) are homologous of the Bcl-2 family of proteins.
FUNDC1	Fun14 domain-containing protein 1
LC3	Light chain 3 protein
Drp1	Dynamin-related protein 1
NLRP3	NOD-, LRR-, and pyrin domain-containing protein 3
TXNIP	Thioredoxin-interacting protein
mtDNA	Mitochondrial DNA
mtROS	Mitochondrial reactive oxygen species
RPE	Retinal pigment epithelium
REC	Retinal endothelial cells
